# Isolation, Biochemical and Molecular Identification, and *In-Vitro* Antimicrobial Resistance Patterns of Bacteria Isolated from Bubaline Subclinical Mastitis in South India

**DOI:** 10.1371/journal.pone.0142717

**Published:** 2015-11-20

**Authors:** P. L. Preethirani, Shrikrishna Isloor, S. Sundareshan, V. Nuthanalakshmi, K. Deepthikiran, Akhauri Y. Sinha, D. Rathnamma, K. Nithin Prabhu, R. Sharada, Trilochan K. Mukkur, Nagendra R. Hegde

**Affiliations:** 1 Department of Microbiology, Karnataka Veterinary Animal and Fisheries Sciences University, Hebbal, Bengaluru, India; 2 Ella Foundation, Genome Valley, Turkapally, Shameerpet Mandal, Hyderabad, India; 3 School of Biomedical Sciences, Faculty of Health Sciences, Curtin University, Bentley, Perth, Western Australia, Australia; Auburn University, UNITED STATES

## Abstract

Buffaloes are the second largest source of milk. Mastitis is a major impediment for milk production, but not much information is available about bubaline mastitis, especially subclinical mastitis. The aim of this study was to (a) investigate the application of various tests for the diagnosis of bubaline subclinical mastitis, (b) identify the major bacteria associated with it, and (c) evaluate the antibiotic resistance pattern of the bacteria. To this end, 190 quarter milk samples were collected from 57 domesticated dairy buffaloes from organized (64 samples) and unorganized (126 samples) sectors. Of these, 48.4%, 40.0%, 45.8%, 61.1%, and 61.6% were positive for subclinical mastitis by somatic cell count, electrical conductivity, California mastitis test, bromothymol blue test, and N-acetyl glucosaminidase test, respectively. As compared to the gold standard of somatic cell count, California mastitis test performed the best. However, a combination of the two methods was found to be the best option. Microbiological evaluation, both by biochemical methods as well as by monoplex and multiplex polymerase chain reaction, revealed that coagulase-negative staphylococci were the most predominant (64.8%) bacteria, followed by streptococci (18.1%), *Escherichia coli* (9.8%) and *Staphylococcus aureus* (7.3%). Most of the pathogens were resistant to multiple antibiotics, especially to β-lactam antibiotics. We propose that California mastitis test be combined with somatic cell count for diagnosis of subclinical mastitis in domestic dairy buffaloes. Further, our results reveal high resistance of the associated bacteria to the β-lactam class of antibiotics, and a possible major role of coagulase-negative staphylococci in causing the disease in India.

## Introduction

Milk and dairy products constitute the major protein source for much of the populace in low to middle income countries. Cattle and buffaloes contribute to an overwhelmingly large proportion of the total milk production worldwide. India is the largest producer and consumer of milk, and buffalo’s milk is produced and consumed at least as much, if not more, as cow’s milk. India contributes to 56% of the total population of buffalo in the world, and 55% of the total milk produced in India is from dairy buffaloes [1]. However, despite genetic upgradation and modern methods of livestock rearing, production per capita has remained low [2]. One reason for this could be mastitis, especially of the subclinical type.

Mastitis remains one of the most common economic problems of dairy industry worldwide. It is mainly caused by bacteria, and the infection results in inflammation and pathophysiological changes in the udder tissue, resulting in compromised milk quality and decreased amount of milk production. Depending on the severity of inflammation, the disease can be categorized into subclinical or clinical [3]. Clinical mastitis can be readily detected whereas asymptomatic conditions make detection of subclinical mastitis (SCM) difficult. Nonetheless, SCM contributes to two-thirds of the economic losses in total milk production [4,5]. Therefore, barn-side tests, when performed routinely, are valuable for timely detection and cure of SCM. The most commonly accepted method for the detection of SCM is the somatic cell count (SCC), which is due to increased influx or shedding of inflammatory and desquamated epithelial cells into milk during mastitis, affecting the quality and yield of milk [6,7]. In addition to SCC, electrical conductivity (EC) of milk, bromothymol blue (BTB) test and California mastitis test (CMT) are also employed to detect SCM. These tests reflect udder infection. Whereas BTB measures the abnormal increase in milk pH, CMT detects the release of nucleic acid subsequent to lysis of somatic cells by using a detergent [8]. Another way of detecting SCM is to measure the activity of the enzyme *N*-acetyl-β-D-glucosaminidase (NAGase), a lysosomal enzyme released into milk during inflammation [9]. Although not without limitations [3], these tests are widely employed across global communities of milk producers. We have also reported on the use of such tests in the detection of bovine SCM [10].

The bacterial mastitogens are classified as either “contagious” or “environmental” [11]. Among these, *Staphylococcus aureus* and *Streptococcus agalactiae* are the important contagious pathogens, and *Escherichia coli*, *Klebsiella pneumoniae*, and *Streptococcus uberis* are the predominant environmental pathogens, whereas *Streptococcus dysgalactiae* can be both an environmental and a contagious pathogen [4,12,13]. Measures such as hygienic and other husbandry practices, including type of stalls, bedding, nutrition, manure removal etc., as well as dry cow therapy are recommended for preventing infections with these pathogens [14,15]. Antibiotics are typically used in dry cow therapy as well as to treat clinical or subclinical mastitis caused by bacterial pathogens. However, irrational use of antibiotics can lead to the emergence of antibiotic resistance, which in turn can compromise cure rates [13,16]. Culling of animals to contain the spread of the disease is not financially viable, and hence, the assessment of antibiogram profile of the strains is the only available option for designing and implementing therapeutic regimen.

Most of the mastitis studies have been conducted in bovines; however, reports from the recent past indicate that domesticated dairy buffaloes are also affected with similar frequency [17–22]. Nevertheless, research on bubaline SCM in India remains scanty [23]. In this context, the present study was conducted to (a) investigate the application of various tests for the diagnosis of bubaline subclinical mastitis, (b) identify the major bacteria associated with it, and (c) evaluate the antibiotic resistance pattern of the bacteria.

## Materials and Methods

### Ethics Statement

According to the guidelines of the Committee for the Purpose of Control and Supervision of Experiments on Animals, which regulates animal experimentation in India, it is not required to obtain permission from or to inform the Institutional Animal Ethics Committee for collection of milk samples from animals. However, oral permission was obtained from the farmers who owned the animals, and requisite results were communicated as desired by the relevant farmers. All samples were collected by veterinarians either involved in the study or working on farms.

### Screening for SCM

A total of 190 bubaline quarter milk samples were collected under aseptic conditions from 57 domesticated dairy buffaloes from two organized farms (n = 64; Dharwad district, Karnataka) and three unorganized sectors (n = 126; in and around Bengaluru district, Karnataka). Care was exercised to disinfect the teat before collecting the sample from each quarter. The samples were transported to the laboratory on ice. The screening for SCM was conducted by SCC, EC, CMT and BTB test using Nucleocounter (M/s ChemoMetec, Denmark), Milk Checker (Oriental Instruments Limited, Tokyo, Japan, or Mastitis Detector, Draminski, Olsztyn, Poland), CMT reagents and BTB strips (Nice Chemicals Pvt. Ltd., Cochin, India), respectively. The cut-off values for SCC and EC for declaring SCM were 2 x 10^5^ cells/mL and 3.8 mS/cm, respectively. The activity of NAGase was measured as described previously [24,25]. Briefly, milk samples were incubated with 4-methylumbelliferyl-*N*-acetyl-β-D-glucosaminide in 0.25 M citrate buffer (pH 4.4) for 5 min at 37°C and analyzed by flurimetry. Enzyme activity of > 6.28 μmoles min^-1^ mL^-1^ (equivalent to the corrected optical density value of 0.09 at 405 nm) was taken as positive index of SCM.

The data were further transformed to calculate diagnostic sensitivity, diagnostic specificity, positive predictive value (PPV), negative predictive value (NPV), and analytical specificity by considering SCC as the gold standard, as follows:

Diagnostic sensitivity=[no.of true positives/(no.of true positives+no.of false negatives)]x100

Diagnostic specificity=[no.of true negatives/(no.of true negatives+no.of false positives)]x100

PPV=[no.of true positives/(no.of true positives+no.of false positives)]x100

NPV=[no.of true negatives/(no.of true negatives+no.of false negatives)]x100

Analytical specificity(accuracy)=[no.of true positives/no.of standard positives]x100

### Reference isolates

The reference isolates for streptococci, *Streptococcus agalactiae* (AD1) (Accession No. HM 355961), *Streptococcus dysgalactiae* (AD3) (Accession No. HC 359248), and *Streptococcus uberis* (AD6) (Accession No. HC 355972), were a kind gift of Dr. Bibek Ranjan Shome, National Institute of Veterinary Epidemiology and Disease Informatics, Bengaluru, India. Confirmed isolates from our collection were used as reference strains for *Staphylococcus aureus* (Accession No. JX298873) and *E*. *coli* (Accession No. JF926686).

### Identification of bacteria by biochemical methods

Milk samples with SCC ≥ 2 X 10^5^ / mL and / or EC ≥ 3.8 mS/cm and / or positive by BTB, CMT or NAGase test were subjected to isolation of staphylococci, streptococci and *E*. *coli* according to previously described protocols [26]. Briefly, the samples were enriched for streptococci and staphylococci by inoculating 1 mL of milk into 5 mL of brain heart infusion (BHI) broth and incubating for 6 h at 37°C. For streptococci, culturing was carried out in an incubator with 5% CO_2_. Initial enrichment for *E*. *coli* was done using tryptone phosphate broth and incubating for 18 h at 37°C. The cultures were then streaked onto mannitol salt agar or blood agar or McConkey’s agar, and incubated for 24, 48 or 72 h for staphylococci, streptococci and *E*. *coli*, respectively. Based on colony morphology, the potential staphylococcal isolates were re-streaked onto BHI agar. Based on hemolytic pattern and colony morphology, the potential streptococcal isolates were re-streaked onto blood agar. Based on lactose fermentation, the potential *E*. *coli* isolates were re-streaked onto eosin methylene blue (EMB) agar, and the resultant metallic sheen colonies were again re-streaked on BHI agar.

Besides Gram’s staining, biochemical characterization of the isolates was performed by following standard procedures as described previously [27]. For staphylococci, catalase, coagulase, thermonuclease, Voges-Proskauer (VP), and urease tests were performed. Catalase, esculin hydrolysis, pyrrolidonyl arylamidase (PYR), sugar fermentation, and hippurate hydrolysis test were employed for the identification of streptococci. For the identification of *E*. *coli*, the indole-methyl red-VP-citrate (IMViC) test was used.

### Identification of bacterial species by PCR

Genomic DNA was extracted using the HiYield™ Genomic DNA Mini Kit (Real Biotech Corporation, Banqiao city, Taiwan) following the manufacturer’s instructions. The *Staphylococcus* and *Streptococcus* genus-specific *tuf* genes as well as the *E*. *coli* species-specific *alr* gene were amplified by monoplex PCR [28,29]. Multiplex PCR for streptococci involved amplification of *sip* and *pau* genes in one tube and that of *16S rRNA* gene in another tube. Multiplex PCR for CoNS involved amplification of four different species-specific products in the first tube and that of five different species-specific products in the second tube, as described previously [30]. In all cases, whether monoplex or multiplex, 1 μg of genomic DNA template was used along with 10 pmol/L of each of the primers, 1 mmol/L of each of the dNTPs and 1 U of *Taq* polymerase (Bangalore Genei, Bengaluru, India) in a total reaction volume of 25 μL. The primers and the annealing conditions are shown in Table 1, and all the reactions were carried out with a denaturation of 95°C/60 s and extension of 72°C/2 min for 35 cycles. The PCR products were analyzed by agarose gel electrophoresis, ethidium bromide staining and UV transillumination.

**Table 1:** Primers, annealing conditions and PCR combinations used in the present study.

Primer set	Organism	Target gene	Primer sequence (5’– 3’)	Product size (bp)	Annealing temperature	Reference
1	Genus *Streptococcus*	*tuf*	CAACTTGACGAAGGTCCTGCA (forward); TGGGTTGATTGAACCTGGTTTA (reverse)	110	50°C	[28]
2	Genus *Staphylococcus*	*tuf*	GAAGAATTATTAGAATTAGT (forward); GTGATTGAGAATACGTCCTCAAC (reverse)	235	50°C	[29]
3	*Staphylococcus aureus*	*nuc*	GTGCTGGCATATGTATGGCAATTGT (forward); TACGCCGTTATCTGTTTGTGATGC (reverse)	181	54°C	[29]
4	*Escherichia coli*	*alr*	CTGGAAGAGGCTAGCCTGGACGAG (forward); AAAATCGCCACCGGTGGAGCGATC (reverse)	369	57°C	[29]
5	*Staphylococcus arlettae*	*gap*	ATCTCTGCTCCAGCATCAGG (forward); AGGAGCGTCTTGTGTGCTTT (reverse)	216	60°C	[30]
6	*Staphylococcus chromogenes*	*sod*A	GTGACTAAGTTAAACGATGCAG (forward); CCATTATTTACAACGAGCCATG (reverse)	303	60°C	[30]
7	*Staphylococcus sciuri*	*gap*	ATTTCAGCTCCAGCATCAGG (forward); TGGAACACGTTGAGCTGATC (reverse)	354	60°C	[30]
8	*Staphylococcus epidermidis*	*rpo*B	AGGGCCTGGTGGATTAACAC (forward); TTGCATGTTTGCTCCCATTA (reverse)	466	60°C	[30]
9	*Staphylococcus saprophyticus*	*gap*	CGTTGACGGAATCGACGTAG (forward); TGCGCTCCTCCATCTAATTT (reverse)	630	60°C	[30]
10	*Staphylococcus equorum*	*sod*A	AACGCTGCAGTTGAAGGAAC (forward); GCAGCTTGGTTAGCAAACTCTTC (reverse)	245	60°C	[30]
11	*Staphylococcus haemolyticus*	*sod*A	GCAGTTGAGGGAACAGATCTTG (forward); CTAACTGACCATTGTTAACTACTAACC (reverse)	282	60°C	[30]
12	*Staphylococcus xylosus*	*rpo*B	GTCTAGTTATGCCCGTGTGAATG (forward); AACAATTGCAGCACCTGAGTC (reverse)	433	60°C	[30]
13	*Staphylococcus simulans*	*gap*	CTACACTAGCGACGAAAAAGCAC (forward); CGTTTACTTCTTCGATTGTTACGTC (reverse)	482	60°C	[30]
14	*Staphylococcus fluerettii*	*rpo*B	ATCAGCTCTTGGACCCGG (forward); GTCACGAGCAGTTACGTGTTCC (reverse)	550	60°C	[30]
15	*Streptococcus agalactiae*	*sip*	CTATTGACATCGACAATGGCAGC (forward); GTTACTGTCAGTGTTGTCTCAGGA (reverse)	266	57°C	[28]
16	*Streptococcus uberis*	*pau*	TGCTACTCAACCATCAAAGGTTGC (forward); TAGCAGTCTCAGTAGGATGAGTGA (reverse)	439	57°C	[28]
17	*Streptococcus dysgalactiae*	*16S rRNA*	GGAGTGGAAAATCCACCAT (forward); CGGTCAGGAGGATGTCAAGAC (reverse)	549	57°C	[28]

Notes:

1 Primer sets 1 to 4 were used in monoplex format to perform genus- (streptococcus, staphylococcus) or species (*E*. *coli*) -specific identification.

2 Primer sets 5 to 14 were used in a 2-tube multiplex format for the identification of staphylococcal species. Sets 5 to 9 and 10 to 14 were used in two separate tubes for this purpose.

3 Primer sets 15 to 17 were used in a 1-tube multiplex format for the identification streptococcal species.

4 Duration of annealing was 30 s in all cases.

*5 alr*: alanine racemase; *gap*: glyceraldehyde-3-phosphate dehydrogenase; *sod*: superoxide dismutase; *nuc*: thermostable nuclease; *pau*: plasminogen activator; *rpo*: RNA polymerase; *sip*: surface immunoglobulin protein; *tuf*: elongation factor Tu.

### Antimicrobial susceptibility testing

To analyze the antibiogram profile of the isolates, disk diffusion method [31] was employed using 15 commercially available antimicrobial sensitivity discs (HiMedia Laboratories, Mumbai). The sensitivity/resistance was interpreted based on the zone of inhibition, inclusive of margins, following the guidelines of the Clinical Laboratory Standards Institute [32].

## Results and Discussion

In order to estimate the extent of SCM in domesticated dairy buffaloes, 190 milk samples collected from 57 animals were subjected to five different tests. It should be noted that the samples correspond to mammary quarters, and not all the quarters were sampled for each animal. All quarters were sampled from 40 animals whereas only one or two quarters were sampled from six animals each, and three quarters were sampled from five animals each (data not shown).

Somatic cell count (SCC) is considered to be the gold standard for ascertaining clinical or subclinical mastitis [9,26]. However, it has been difficult to define the threshold for SCC which would be indicative of mastitis since SCC can overlap between mastitis affected and unaffected animals or even between udder quarters of the same animal [33,34]. Moreover, multiple factors such as stage of lactation, age, breed, parity, season, stress and diurnal variations can also influence SCC [34]. When the 190 milk samples that we collected were subjected to SCC analysis, 24.2%, 18.9%, 11.6%, and 45.3% were grouped under 0–0.5 x 10^5^, 0.5–1 x 10^5^, 1–2 x 10^5^ and >2 x 10^5^ cells/mL, respectively (Table 2, and data not shown). In case of buffaloes, SCC of 2 x 10^5^ cells/mL has been considered a cut-off for declaring SCM [19,35,36]. Accordingly, 86 samples (45.3%) could be declared as SCM positive. Interestingly, the rate of SCM was not very different between organized farms (46.9%) and unorganized sectors (44.4%) (Table 2).

**Table 2:** Diagnosis of subclinical bubaline mastitis by various tests [data are presented as number (%) of quarter milk samples].

Setting	Farm/Sector	SCC	EC	CMT	BTB test	NAGase test
Organized	Farm A (n = 20)	6 (30.0)	17 (85.0)	3 (15.0)	9 (45.0)	4 (20.0)
Organized	Farm B (n = 44)	24 (54.5)	13 (29.5)	21 (47.7)	24 (54.5)	31 (70.5)
**Total for organized farms**	**2 farms (n = 64)**	**30 (46.9)**	**30 (46.9)**	**24 (37.5)**	**33 (51.6)**	**35 (54.7)**
Unorganized (Village)	Sector A (n = 32)	4 (12.5)	31 (96.9)	4 (12.5)	11 (34.4)	12 (37.5)
Unorganized (Village)	Sector B (n = 32)	19 (59.4)	4 (12.5)	18 (56.3)	23 (71.9)	23 (71.9)
Unorganized (Village)	Sector C (n = 62)	33 (53.2)	11 (17.7)	41 (66.1)	49 (79.0)	47 (75.8)
**Total for unorganized sectors**	**3 sectors (n = 126)**	**56 (44.4)**	**46 (36.5)**	**63 (50.0)**	**83 (65.9)**	**82 (65.1)**
**Grand total**	**2 farms and 3 villages**	**86 (45.3)**	**76 (40.0)**	**87 (45.8)**	**116 (61.1)**	**117 (61.6)**
**Sensitivity**		**NA**	**38.4**	**83.7**	**80.2**	**93.1**
**Specificity**		**NA**	**58.7**	**85.6**	**54.8**	**66.0**
**Positive predictive value**		**NA**	**43.4**	**82.8**	**59.5**	**69.8**
**Negative predictive value**		**NA**	**53.5**	**86.4**	**77.0**	**91.9**
**Analytical specificity**		**NA**	**38.4**	**83.7**	**80.2**	**94.2**

SCC = somatic cell count; EC = electrical conductivity; CMT = California mastitis test; BTB–bromothymol blue; NAGase = N-acetyl-β-D-glucosaminidase; NA = not applicable

Cut-off values used for declaring positivity were 2 x 10^5^ / mL of milk for SCC, ≥ 3.8 mS / cm for EC and corrected OD_405_ value of 0.09 for NAGase test, as described in materials and methods. CMT and BTB are qualitative tests and hence do not have cut-offs.

In addition to SCC, electrical conductivity (EC) of milk, BTB test and CMT were also evaluated to determine the extent of SCM. By CMT and BTB test, 45.8% and 61.1% of the samples were positive, respectively (Table 2). There are very few reports describing the application of the measurement of EC for detection of mastitis in buffaloes, and a break point value of 3.8 mS/cm has been suggested [37]. Accordingly, 76 milk samples (40%) were positive. When the activity of NAGase was analyzed, 61.6% of the samples could be declared as originating from SCM (Table 2). When diagnostic and analytical parameters were calculated, CMT as well as BTB and NAGse tests showed high diagnostic sensitivity whereas only CMT showed high diagnostic specificity. On the other hand, the ability to predict positive cases was high only for CMT, whereas both CMT and NAGase test were fairly reliable in predicting negative cases. Analytical specificity was good for CMT and BTB test, and best for NAGase test. Measuring EC was by far the least reliable method as the values for all the diagnostic and analytical parameters were <60% (Table 2). Overall, the best performance was with CMT followed by NAGase. Given that both the gold standard test of SCC and NAGase test require additional laboratory equipment for performing these tests, our results suggest that CMT could be reliably used in place of SCC as a barn-side diagnostic test for declaring SCM in domestic dairy buffaloes. One, however, needs to bear in mind that factors other than mastitis can influence the results [3], and therefore a combination of these tests rather than one single test may be necessary for accurate diagnosis of SCM. In this regard, it is noteworthy that 15 samples were positive by CMT but negative by SCC, and 14 samples were positive by SCC but negative by CMT (data not shown), suggesting 29 more samples could have been declared as positive if a combination of CMT and SCC were employed.

To identify the bacteria associated with SCM, the 92 milk samples which were positive in any of the aforementioned tests were subjected to isolation and identification. A total of 195 bacterial isolates were obtained by standard biochemical procedures [27]. Mixed infections with one or more of *S*. *aureus*, CoNS, *E*. *coli* and streptococci were common, and were observed with 70 milk samples, resulting in 195 isolates from 92 samples. None of the samples were negative for growth. Biochemical tests revealed that CoNS were the major pathogens (n = 125), followed by streptococci (n = 35), *E*. *coli* (n = 21) and *S*. *aureus* (n = 14) (Table 3). Two isolates which were otherwise characteristic of *E*. *coli* were negative by the indole test.

**Table 3:** Comparison of methods for the identification of bacteria associated with subclinical bubaline mastitis (data are presented as number of isolates).

Farm/Sector	*Streptococcus* spp.		*Staphylococcus aureus*		Coagulase-negative staphylococci		*E*. *coli*	
	Biochemical	PCR	Biochemical	PCR	Biochemical	PCR	Biochemical	PCR
Farm A	14	14	2	2	6	6	18	16
Farm B	0	0	9	9	46	46	0	0
Sector A	1	1	0	0	10	10	0	0
Sector B	5	5	3	3	26	26	0	0
Sector C	15	15	0	0	37	37	3	3
**Total**	**35**	**35**	**14**	**14**	**115**	**115**	**21**	**19**

**Fig 1:**
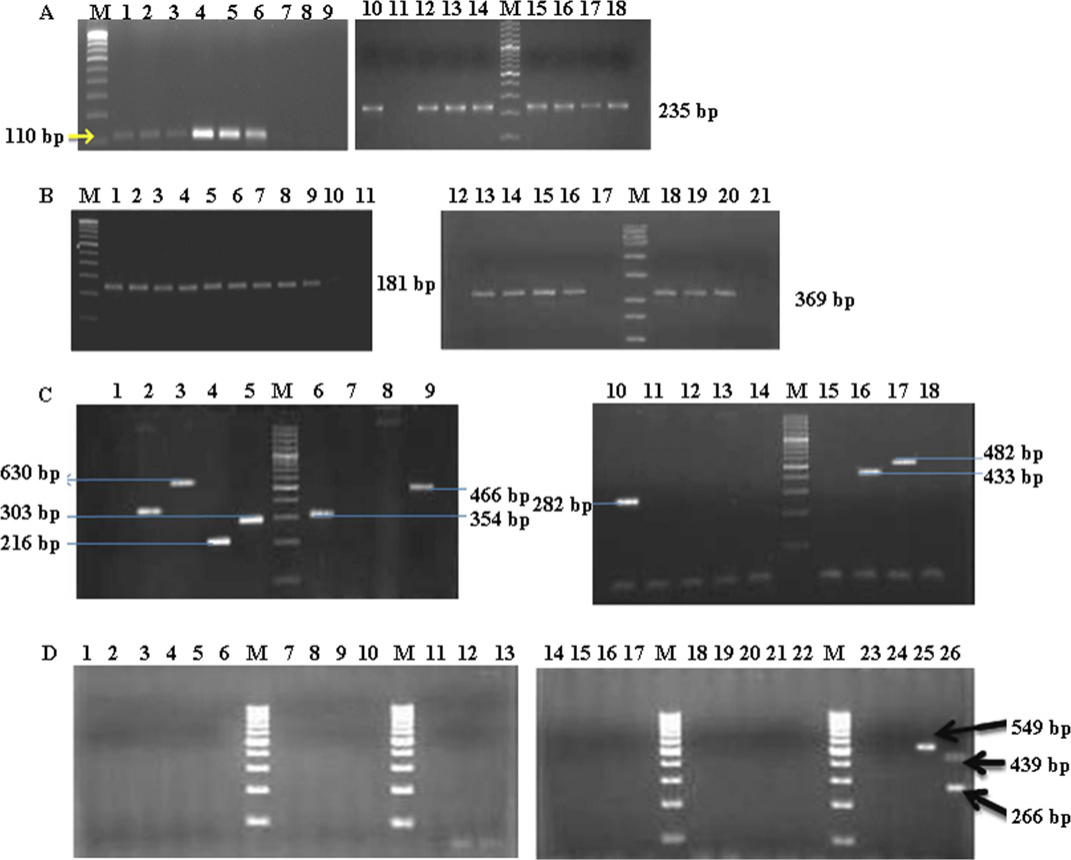
Polymerase chain reaction for the identification of bacteria. Genomic DNA was isolated from the obtained isolates as well as reference strains, and subjected to mono- or multi-plex PCR as described in the Materials and Methods and Table 1. The experiments were repeated at least three times and representative gel pictures are shown. Note that each panel is composed from two separate gels since all the samples could not be accommodated in a single gel. **(A) PCR for genus-specific *tuf* genes of streptococci and staphylococci.** Lane designation: M, 100 bp ladder; 1–5, *Streptococcus* spp. isolates; 6, Reference strain Streptococcus AD1; 7, No template control for streptococcus; 8, Negative control (*S*. *aureus*, *E*. *coli*); 9, Reagent control; 10, Reference strain *S*. *aureus* 96; 11, No template control for staphylococcus; 12–18: *Staphylococcus* spp. isolates. **PCR for *S*. *aureus nuc* (lanes 1–11) and *E*. *coli alr* (lanes 12–21) genes.** Lane designation: M, 100 bp ladder; 1–8, *S*. *aureus* test isolates; 9, Reference strain SAU-3; 10, Negative control (*E*. *coli*); 11, No template control; 12, Negative control (*S*. *aureus*); 13, Reference strain EC11 (*E*. *coli*); 14–16, Test isolates of *E*. *coli*; 17, No template control; 18–20, Test isolates; 21, Negative control (streptococcus). **(B) PCR for the identification of CoNS species.** Lane designation: M, 100 bp ladder; 1, *S*. *haemolyticus* (MTCC 3383) control; 2, *S*. *sciuri* (MTCC 6154) control; 3, *S*. *saprophyticus* (MTCC 6155) control; 4, *S*. *arlettae* (JQ764624) control; 5, *S*. *chromogenes* (MTCC 3545) control; 6, *S*. *sciuri* (MTCC 6154) control; 7, *S*. *xylosus* (FJ90627.1) control; 8, *S*. *simulans* (AF495498.1) control; 9, *S*. *epidermidis* (MTCC 3615) control; 10, *S*. *haemolyticus* (MTCC 3383) control; 11, *S*. *sciuri* (MTCC 6154) control; 12, *S*. *saprophyticus* (MTCC 6155) control; 13, *S*. *arlettae* (JQ764624) control; 14, *S*. *chromogenes* (MTCC 3545) control; 15, *S*. *sciuri* (MTCC 6154) control; 16, *S*. *simulans* (AF495498.1) control; 17, *S*. *xylosus* (FJ90627.1) control; 18, *S*. *epidermidis* (MTCC 3615) control. This Panel represents two mutually exclusive pictures depicting the results of the standardization of one tube each of the two-tube multiplex PCR. In the left panel, primers for *S*. *arlettae*, *S*. *chromogenes*, *S*. *sciuri*, *S*. *epidermidis* and *S*. *saprophyticus* were used, and *S*. *haemolyticus*, *S*. *xylosus* and *S*. *simulans* DNA served as negative controls. In the right panel, primers for *S*. *equorum*, *S*. *haemolyticus*, *S*. *xylosus*, *S*. *simulans* and *S*. *fluerettii* were used, and *S*. *sciuri*, *S*. *sapryphyticus*, *S*. *arlettae*, *S*. *chromogenes* and *S*. *epidermidis* DNA served as negative controls. Numbers in parentheses indicate the GenBank Accession numbers or the MTCC culture designations. **(C) PCR for the identification of *Streptococcus* species.** Lane designation: M, 100 bp ladder; 1–20, Test streptococcal isolates streptococci (no amplification); 21, Negative control (*S*. *aureus*); 22, Negative control (*E*. *coli*); 23 & 24, No template control; 25, Tube 2 positive control (*Streptococcus* reference strain AD3); 26, Tube 1 positive controls (*Streptococcus* reference strains AD1 and AD6).

Biochemical identification of the bacteria was corroborated by PCR (Fig 1). Initially, the *tuf* gene was targeted for differentiating streptococci and staphylococci at the genus level [38]. Expectedly, all the biochemically identified streptococci and staphylococci were confirmed as such with the expected amplicon sizes of 110 bp and 235 bp, respectively (Fig 1A, left and right panels, respectively). Biochemical identification of *E*. *coli* was confirmed by PCR for *alr*, an essential gene for cell wall synthesis [39,40]. Notably, the 369 bp product (Fig 1B, right panel) was only observed with 19 of the 21 presumed *E*. *coli* isolates, and not with the two isolates that were negative by the indole test.

The overall prevalence of staphylococci, streptococci and *E*. *coli* was 72.0%, 18.1% and 9.8%, respectively (Table 2). This distribution was similar to those reported previously in India [26,41]. Surprisingly, among staphylococci (n = 125), 89.9% of the isolates were CoNS (Table 2). This is in contrast to the situation with bovine mastitis where up to 58% of the SCM in India has been reported to be due to *S*. *aureus* [42,43], but similar to observations in dairy cattle in some countries such as Uganda [44], as well as in modern cattle dairy farms where contagious pathogens have been controlled [45–48]. Higher prevalence of CoNS has also been reported for ovine or caprine mastitis in China, Iran, Israel, Italy, the Netherlands, New Zealand, Spain, and Sweden [49–59]. Whereas all these studies, including ours, suggest a potential role for CoNS in causing SCM, there is as yet no concrete evidence for a cause and effect relationship between the two [47,48].

Biochemical differentiation of *S*. *aureus* and CoNS was confirmed by the amplification of the *nuc* gene [60], where a 181 bp amplicon was observed with *S*. *aureus* (Fig 1B, left panel). To identify which of the CoNS species were involved in bubaline SCM, a two-tube PCR was performed (Fig 1C), where 91 of the 125 isolates yielded positive amplification. Further analyses revealed that these 91 isolates comprised of eight species *viz*., *S*. *chromogenes* (n = 30), *S*. *epidermidis* (n = 28), *S*. *sciuri* (n = 9), *S*. *xylosus* (n = 7), *S*. *haemolyticus* (n = 6), *S*. *arletti* (n = 5), *S*. *simulans* (n = 5), and *S*. *saprophyticus* (n = 1). The rest of the 34 isolates could not be identified to species level by the PCR employed. The presence of *S*. *epidermidis* and *S*. *chromogenes* as the predominant species among the CoNS was in accordance with other observations reported for bovine mastitis [48,49].

The precise identification of streptococci to the species level could not be achieved even after a thorough biochemical as well as PCR-based characterization. Other researchers have also reported on the inability to differentiate between typical and atypical streptococci by biochemical methods [26,61]. *Streptococcus uberis*, which is characteristically negative for CAMP (Christie-Atkins-Munch-Petersen) test, has been reported to be CAMP test positive by a few researchers [61–63]. Furthermore, Bosshard et al [64] were unable to identify streptococcus to the species level even after using the API 20 Strep system and molecular-based assays. In our experiments, the two-tube multiplex PCR failed to yield an amplicon with any of the primers (Fig 1D). These results suggest that a more detailed analysis of the genomes of *Streptococcus* spp. is required for developing methods to identify and differentiate the species within the genus. A system based on *16S rRNA* may be more useful for this purpose [65].

As antibiotic-resistant pathogens pose a major challenge in treatment of mastitis, we analyzed the antibiogram profile of all the 195 isolates. Among the staphylococci, CoNS were highly resistant to methicillin, amoxycillin/sulbactam and penicillin-G (72% to 85.6%), and displayed intermediate resistance to ceftriaxone/sulbactam, cefoxitin and cefotaxime (48.8% to 52.8%) while they were least resistant to co-trimoxazole, chloramphenicol and gentamicin (3.2% to 4.0%) (Table 4). On the other hand, *S*. *aurues* isolates were most resistant to cefoxitin (100%) followed by penicillin-G and ceftriaxone/sulbactam (both 85.7%), displayed low resistance to co-trimoxazole (7.1%) and oxacillin (21.4%) and intermediate resistance to enrofloxacin (57.1%), and were all susceptible to chloramphenicol (Table 4). These observations suggested that the majority of the staphylococci (CoNS as well as *S*. *aureus*) were resistant to β-lactam class of antibiotics with the exception of oxacillin. These results are in concordance with the previous reports on antibiotic resistance of buffalo mastitis-associated staphylococci [66,67].

**Table 4:** Antibiotic resistance pattern of bacteria [n (%) of isolates] associated with bubaline mastitis.

Antibiotic	Content per disk	Breakpoint to declare resistance	CoNS (n = 125)	*S*. *aureus* (n = 14)	Streptococcus (n = 35)	*E*. *coli* (n = 19)
MET	5 μg	9 mm	107 (85.6)	11 (78.6)	35 (100)	19 (100)
AMS	30/15 μg	31 mm	103 (82.4)	11 (78.6)	30 (85.7)	19 (100)
P	10 μg	28 mm	90 (72)	12 (85.7)	31 (88.6)	19 (100)
AMP	10 μg	28 mm	87 (69.6)	10 (71.4)	29 (82.9)	19 (100)
CIS	30/15 μg	23 mm	66 (52.8)	13 (92.9)	26 (74.3)	19 (100)
CX	30 μg	24 mm	61 (48.8)	14 (100)	31 (88.6)	19 (100)
CTX	30 μg	22 mm	61 (48.8)	12 (85.7)	26 (74.3)	19 (100)
S	10 μg	16 mm	60 (48)	11 (78.6)	32 (91.4)	19 (100)
CTR	30 μg	13 mm	56 (44.8)	10 (71.43)	16 (45.7)	8 (42.1)
EX	10 μg	21 mm	14 (11.2)	8 (57.1)	27 (77.1)	9 (47.4)
AK	10 μg	14 mm	11 (8.8)	5 (35.7)	29 (82.9)	19 (100)
OX	1 μg	10 mm	7 (5.6)	3 (21.4)	10 (28.6)	18 (94.73)
GEN	10 μg	12 mm	5 (4)	5 (35.7)	19 (54.3)	14 (73.68)
C	30 μg	12 mm	5 (4)	0 (0)	5 (14.29)	0 (0)
COT	25 μg	10 mm	4 (3.2)	1 (7.1)	13 (37.14)	2 (10.5)

AK: Amikacin; AMP: Ampicillin; AMS: Amoxycillin with sulbactum; C: Chloramphenicol; CIS: Ceftriaxone with sulbactum; COT: Co-trimoxazole; CTR: Ceftriaxone; CTX: Cefotaxime; CX: Cefoxitin; EX: Enrofloxacin; GEN: Gentamicin; MET: methicillin; OX: Oxacillin; P: Penicillin; S: Streptomycin

Of note, all the *S*. *aureus* isolates showed resistance to cefoxitin whereas only 48.8% of the CoNS were resistant to the antibiotic (Table 4). Cefoxitin and oxacillin have both been used to infer methicillin resistance. However, proportion of *S*. *aureus* and CoNS isolates resistant to oxacillin was 21.4% and 5.6% and that to methicillin was 78.6% and 85.6%, respectively. While the discrepancy between the results for methicillin, oxacillin and cefoxitin is perplexing, it is known that cefoxitin is more sensitive than oxacillin in detecting methicillin resistance [68–71]. Our data with cefoxitin suggests that a high proportion of the staphylococcal isolates, especially *S*. *aureus*, from domesticated dairy buffaloes in India are methicillin-resistant. Other studies in dairy buffaloes from Brazil [72], Egypt [73], India [74], and Iraq [75,76] have reported methicillin resistant *S*. *aureus* (MRSA) ranging from 5.4% to 100% of the tested isolates by employing oxacillin disk diffusion test [72,74–76] or the presence of *mec*A gene [73]. Although not well explored, methicillin-resistant CoNS (MR-CoNS) have also been reported from dairy buffaloes in India and Turkey [77,78]. The high level of prevalence of MRSA as well as MR-CoNS among dairy buffaloes could be of serious public health concern, and therefore needs to be further investigated.

Antibiogram profile of streptococci revealed that these isolates displayed 100% resistance to methicilin and were highly resistant to streptomycin, cefoxitin and penicillin-G (88.6% to 91.4%). They showed intermediate resistance to gentamicin and ceftriaxone (45.0% and 54.3%, respectively), and were least resistant to chloramphenicol and oxacillin (14.3% and 28.6%, respectively) (Table 4). Whereas chloramphenicol sensitivity has been recorded by other researchers [79,80], in contrast to the study of Dhakal *et al* [79], streptococci were highly resistant to β-lactam class of antibiotics (with the exception to oxacillin) as well as fluoroquinolones such as enrofloxacin (Table 4).

*Escherichia coli* isolates showed 100% resistance for amikacin, amoxycillin/sulbactam, ampicillin, cefotaxime, cefoxitin, ceftriaxone/sulbactam, methicillin, penicillin-G and streptomycin. Moreover, all the isolates except one were resistant to oxacillin. They displayed intermediate resistance to enrofloxacin (47.4%), low resistance to cotrimazole (10.5%), and were completely sensitive to chloramphenicol. Alarmingly, resistance to cephalosporins such as cefotaxime, cefoxitin along with penicillin-G and other β-lactam antibiotics suggest that all the isolates produced extended spectrum β lactamases.

Taken together, antibiogram profile of bacteria associated with bubaline SCM suggests high resistance to β-lactam antibiotics, corroborating several other studies on bovine and bubaline mastitis in India [34,81–84]. The high antibiotic resistance could be due to indiscriminate and injudicious use of antibiotics, about which there is no authentic data available even for cattle in India. Increased antibiotic usage is known to increase antibiotic resistance among bacteria isolated from cases of mastitis [85]. Incidentally, considerable amount of antimicrobials are used in India for food animal production, although much of it is probably used in poultry and piggery sectors; in addition, the country is projected to witness a manifold increase in antimicrobial usage in the near future [86]. The antibiotic resistance pattern is likely to be compounded by similar indiscriminate use of antibiotics by humans. Since the large majority of the livestock sector in India is in unorganized form, contribution of human-animal interface to antibiotic resistance of mastitis-associated bacteria cannot be ruled out. In any case, the findings of high antibiotic resistance stress the importance of measures to prevent udder infections.

Our report presents the most detailed study of SCM in domestic dairy buffaloes to date, comprising of evaluation of field diagnostic tests, isolation and identification of the major bacterial pathogens, and antibiogram studies. The prevalence of SCM in domesticated dairy buffaloes of South India was similar in organized and unorganized sectors, and a combination of CMT and SCC might be the best option for diagnosis. Etiologically, CoNS were the predominant organisms associated with SCM in domesticated dairy buffaloes. However, identification of bacteria by biochemical tests may need to be supported by molecular methods. Finally, not only a high proportion of the bacteria were resistant to many individual antibiotics, including methicillin, but many individual isolates were also resistant to multiple antibiotics, indicating that judicious use of antibiotics based on antibiogram testing is required for mastitis therapy.

## Supporting Information

S1 Table Results of subclinical mastitis testing on individual samples by employing various tests. Note: Where indicated, LF, RF, LH and RH refer to left fore, right fore, left hind and right hind quarter sample, respectively. In some cases, the four different quarters are simply labeled as a, b, c and d. The name of the village and farm have not been disclosed for confidentiality.
(DOCX)
