# Correction: Isolation, Biochemical and Molecular Identification, and *In-Vitro* Antimicrobial Resistance Patterns of Bacteria Isolated from Bubaline Subclinical Mastitis in South India

**DOI:** 10.1371/journal.pone.0145897

**Published:** 2015-12-23

**Authors:** The PLOS ONE Staff

## Notice of Republication

Figure 1 was published in error. The error was corrected in the HTML and PDF versions of this article on December 8, 2015, and the corrected figure is available in the supporting information of this article.

## Supporting Information

S1 Fig Corrected Fig 1. (TIF)
