# Supplementary material for: Isolation, Biochemical and Molecular Identification, and In-Vitro Antimicrobial Resistance Patterns of Bacteria Isolated from Bubaline Subclinical Mastitis in South India
Source: PLoS One. 2015 Nov 20;10(11):e0142717. doi: 10.1371/journal.pone.0142717 (PMC4654528; doi:10.1371/journal.pone.0142717)
Supplement: S1 Table — Note: Where indicated, LF, RF, LH and RH refer to left fore, right fore, left hind and right hind quarter sample, respectively. In some cases, the four different quarters are simply labeled as a, b, c and d. The name of the village and farm have not been disclosed for confidentiality. (DOCX) [file pone.0142717.s001.docx]

**S1 Table. Results of subclinical mastitis testing on individual samples**

| **Sample ID** | **SCC (×10^5^/mL)** | **EC (mS/cm)** | **CMT** | **BTB Test** | **NAGase Test (OD value)** |
| --- | --- | --- | --- | --- | --- |
| **Organized farm A (20 samples)** | | | | | |
| 11LF | <10 | 4.1 | - | + | 0.134 |
| 11RF | 0.74 | 4.9 | - | - | 0.129 |
| 11LH | 0.32 | 4.7 | - | - | 0.132 |
| 11RH | 3.03 | 4.9 | + | ++ | 0.208 |
| 28RF | 0.35 | 4.3 | - | + | 0.129 |
| 28LF | 0.24 | 4.1 | - | - | 0.107 |
| 44RH | 0.72 | 4 | - | + | 0.136 |
| 44LH | 0.21 | 3.9 | - | - | 0.121 |
| 44RF | 0.66 | 4.1 | - | - | 0.161 |
| 44LF | <10 | 3.8 | - | - | 0.102 |
| 49LH | 0.17 | 4 | - | + | 0.139 |
| 70RF | 0.18 | 4.2 | - | - | 0.128 |
| 70LF | <10 | 4 | - | + | 0.119 |
| 71LH | <10 | 4.3 | - | + | 0.131 |
| 71RH | 0.19 | 4.8 | - | - | 0.135 |
| G1 | 0.32 | 3.7 | - | - | 0.121 |
| 79RF | 0.24 | 4 | - | + | 0.134 |
| 79RH | 0.95 | 3.2 | + | ++ | 0.182 |
| 86RH | 0.85 | 3.2 | + | - | 0.172 |
| 86LH | <10 | 3.8 | - | - | 0.103 |
| **Organized farm B (44 samples)** | | | | | |
| 1a | 7.87 | 4.5 | + | + | 0.357 |
| 1b | 7.89 | 4.9 | ++ | + | 0.34 |
| 1c | 6.63 | 3.9 | + | - | 0.38 |
| 1d | 2.68 | 2.4 | - | - | 0.24 |
| 2a | 8.05 | 3.2 | + | ++ | 0.385 |
| 2b | 2.43 | 2.1 | + | - | 0.21 |
| 2c | 1.09 | 2.9 | - | + | 0.145 |
| 2d | 2.37 | 2.7 | - | - | 0.197 |
| 3a | 13.43 | 4.9 | +++ | ++ | 0.389 |
| 3b | 16.12 | 5.1 | ++ | + | 0.41 |
| 3c | 15.97 | 4.8 | +++ | + | 0.399 |
| 4a | 14.85 | 4.9 | +++ | ++ | 0.374 |
| 4b | 0.51 | 1.9 | - | - | 0.123 |
| 4c | 8.29 | 3 | + | - | 0.235 |
| 4d | 4.39 | 3.2 | - | + | 0.298 |
| 5a | 2.63 | 2.9 | - | - | 0.189 |
| 5b | 0.84 | 1.4 | - | + | 0.104 |
| 5c | 0.88 | 1.9 | - | + | 0.121 |
| 5d | 0.99 | 2.6 | - | - | 0.187 |
| 6a | 0.45 | 1.8 | - | + | 0.134 |
| 6b | 0.9 | 1.3 | - | + | 0.15 |
| 6c | 0.28 | 2.5 | - | + | 0.121 |
| 7a | 2.8 | 2.8 | - | - | 0.165 |
| 7b | 1.15 | 4.5 | + | - | 0.152 |
| 7c | 6.19 | 4.9 | + | + | 0.32 |
| 7d | 5.6 | 3.5 | + | ++ | 0.301 |
| 8a | 1.86 | 2.7 | - | - | 0.147 |
| 8b | 0.37 | 2.9 | - | + | 0.106 |
| 8c | 1.54 | 1.4 | - | - | 0.126 |
| 8d | 0.8 | 1.8 | - | + | 0.131 |
| 9a | 1.14 | 2.8 | + | + | 0.178 |
| 9b | 2.55 | 3.2 | + | - | 0.179 |
| 9c | 0.9 | 2.5 | - | - | 0.149 |
| 9d | 0.35 | 1.7 | - | + | 0.135 |
| 10a | 7.89 | 3.6 | ++ | ++ | 0.305 |
| 10b | 0.36 | 2.7 | - | - | 0.132 |
| 10c | 2.87 | 2.3 | + | + | 0.241 |
| 10d | 8.98 | 4.2 | ++ | - | 0.361 |
| 11a | 1.65 | 2.6 | - | - | 0.136 |
| 11b | 0.96 | 1.9 | - | - | 0.124 |
| 11c | 3.65 | 3.4 | + | - | 0.232 |
| 11d | 1.64 | 4.6 | - | + | 0.124 |
| 12a | 5.25 | 5 | + | + | 0.237 |
| 12b | 14.38 | 4.7 | ++ | - | 0.398 |
| **Unorganized sector A (32 samples)** | | | | | |
| 1LF | 1.09 | 1.7 | + | + | 0.216 |
| 1LH | 3.42 | 2 | + | + | 0.217 |
| 1RH | 1.05 | 2.3 | + | + | 0.219 |
| 1RF | 0.68 | 1.9 | - | + | 0.142 |
| 2RH | 0.24 | 1.8 | - | - | 0.112 |
| 2RF | 0.32 | 1.9 | - | - | 0.135 |
| 2LH | 0.58 | 1.8 | - | + | 0.153 |
| 2LF | 0.24 | 1.8 | - | - | 0.113 |
| 3LF | 2.39 | 2.5 | + | + | 0.187 |
| 3LH | 12.16 | 2.6 | + | ++ | 0.355 |
| 3RF | 3.49 | 3 | + | ++ | 0.291 |
| 3RH | 3.18 | 2.4 | + | ++ | 0.27 |
| 4RF | 0.5 | 2.1 | - | - | 0.141 |
| 4LH | 0.15 | 2.3 | - | ++ | 0.12 |
| 4RH | 14.07 | 2.3 | + | ++ | 0.387 |
| 4LF | 0.22 | 2 | - | - | 0.128 |
| 5LH | 15.64 | 3.3 | +++ | ++ | 0.391 |
| 5LF | 12.81 | 3.1 | ++ | ++ | 0.353 |
| 5RH | 6.84 | 3.1 | + | ++ | 0.313 |
| 5RF | 0.4 | 2.2 | - | - | 0.134 |
| 6LH | 0.59 | 3.8 | - | - | 0.14 |
| 6RF | 2.15 | 2.8 | +++ | + | 0.168 |
| 6RH | 6.64 | 2.2 | ++ | ++ | 0.301 |
| 6LF | 4.31 | 4.5 | +++ | ++ | 0.2 |
| 7RH | 5.04 | 2.6 | ++ | ++ | 0.295 |
| 7RF | 3.33 | 2.3 | + | + | 0.28 |
| 8LH | 2.05 | 1.8 | - | + | 0.265 |
| 8LF | 0.72 | 3.7 | - | - | 0.165 |
| 9RF | 8.88 | 2.9 | - | ++ | 0.349 |
| 9RH | 11.36 | 3.9 | - | ++ | 0.4 |
| 9LH | 15 | 2.4 | +++ | ++ | 0.398 |
| 9LF | 15.21 | 4 | +++ | - | 0.37 |
| **Unorganized sector B (32 samples)** | | | | | |
| 1LF | 0.53 | 4.3 | - | - | 0.12 |
| 1LH | 0.54 | 4.3 | - | - | 0.134 |
| 1RF | 0.44 | 4.2 | - | + | 0.104 |
| 1RH | 0.78 | 4.3 | - | - | 0.168 |
| 2LF | 0.52 | 5 | - | + | 0.165 |
| 2LH | 1.36 | 4.5 | - | - | 0.198 |
| 2RF | 0.55 | 5.1 | - | ++ | 0.136 |
| 2RH | 1.33 | 4.1 | - | - | 0.183 |
| 3LF | 0.14 | 4.2 | - | - | 0.101 |
| 3LH | 0.13 | 4.2 | - | - | 0.12 |
| 3RF | 0.27 | 4.1 | - | - | 0.104 |
| 3RH | 0.23 | 4.1 | - | - | 0.14 |
| 4LF | 8.88 | 4.7 | + | ++ | 0.298 |
| 4LH | 7.79 | 4.6 | + | + | 0.31 |
| 4RF | 9.43 | 4.6 | + | ++ | 0.34 |
| 4RH | 8.58 | 4.5 | + | ++ | 0.313 |
| 5LF | 0.55 | 4.3 | - | - | 0.14 |
| 5LH | 0.41 | 4.3 | - | - | 0.154 |
| 5RF | 0.18 | 4.7 | - | - | 0.103 |
| 5RH | 0.2 | 4.4 | - | + | 0.14 |
| 6LF | <0.1 | 4.4 | - | - | 0.12 |
| 6LH | <0.1 | 4 | - | - | 0.13 |
| 6RF | 0.18 | 3.4 | - | - | 0.14 |
| 6RH | 0.15 | 4.3 | - | ++ | 0.132 |
| 7LF | 0.17 | 4.6 | - | - | 0.125 |
| 7LH | 0.44 | 4.5 | - | + | 0.163 |
| 7RF | 0.31 | 4.6 | - | - | 0.128 |
| 7RH | 0.17 | 4.3 | - | - | 0.125 |
| 8LF | 0.28 | 4.2 | - | + | 0.134 |
| 8LH | 0.68 | 4.3 | - | - | 0.169 |
| 8RF | 0.35 | 4.1 | - | - | 0.142 |
| 8RH | 0.8 | 4.4 | - | - | 0.159 |
| **Unorganized sector C ( 62 samples)** | | | | | |
| 10RF | 0.71 | 2.2 | - | + | 0.14 |
| 10LF | 0.81 | 2.2 | - | + | 0.16 |
| 10LH | 1.49 | 2.3 | - | + | 0.189 |
| 10RH | 3.51 | 2.6 | + | ++ | 0.21 |
| 11RH | 0.77 | 1.6 | - | + | 0.16 |
| 11LH | 0.48 | 2.4 | - | - | 0.11 |
| 11LF | 0.31 | 2.3 | - | - | 0.102 |
| 11RF | 11.11 | 2.4 | + | - | 0.324 |
| 12RF | 6.15 | 4.4 | ++ | ++ | 0.3 |
| 12RH | 2.17 | 4 | + | - | 0.276 |
| 12LH | 3.17 | 3.7 | + | + | 0.234 |
| 12LF | 5.61 | 3.2 | + | ++ | 0.32 |
| 13RF | 0.5 | 2.8 | - | - | 0.14 |
| 13LF | 0.46 | 2.2 | - | - | 0.135 |
| 13RH | 0.36 | 2.2 | - | - | 0.124 |
| 14LH | 11.87 | 5.3 | +++ | ++ | 0.313 |
| 14LF | 10.62 | 5.8 | +++ | ++ | 0.32 |
| 14RH | 8.17 | 4.5 | ++ | ++ | 0.299 |
| 14RF | > 20 | 8.4 | - | ++ | 0.42 |
| 15LF | 2.28 | 2.3 | + | - | 0.21 |
| 15LH | 1.8 | 2.5 | + | ++ | 0.198 |
| 15RF | 1.53 | 2.3 | + | ++ | 0.178 |
| 15RH | 1.92 | 2.4 | + | ++ | 0.176 |
| 16LH | 1.5 | 2.9 | + | ++ | 0.13 |
| 16LF | 10.7 | 3.6 | ++ | ++ | 0.34 |
| 16RF | 6.52 | 3.9 | + | ++ | 0.314 |
| 16RH | 1.82 | 4.6 | + | ++ | 0.132 |
| 17LH | 12.23 | 3.6 | + | ++ | 0.378 |
| 17RH | 12.94 | 3.3 | +++ | ++ | 0.345 |
| 17LF | 8.32 | 3.9 | + | ++ | 0.312 |
| 17RF | 11.65 | 3.7 | +++ | ++ | 0.334 |
| 18LH | 0.42 | 2.5 | - | ++ | 0.132 |
| 18RF | 1.26 | 3.5 | - | ++ | 0.198 |
| 18LF | 0.5 | 1.6 | - | ++ | 0.13 |
| 19LH | 0.27 | 1.9 | - | ++ | 0.143 |
| 19RH | 1.58 | 1.4 | + | ++ | 0.167 |
| 19RF | 0.89 | 2.7 | + | ++ | 0.139 |
| 20LH | 8.45 | 2 | + | ++ | 0.376 |
| 20LF | 5.25 | 3.6 | +++ | ++ | 0.34 |
| 20RF | 0.91 | 2.6 | - | ++ | 0.154 |
| 18RH | 0.37 | 2.4 | - | ++ | 0.14 |
| 21RF | 3.43 | 3.2 | + | ++ | 0.28 |
| 22LF | 4.62 | 1.6 | + | ++ | 0.299 |
| 23RF | 3.8 | 2.8 | + | ++ | 0.287 |
| 23LF | 0.94 | 1.4 | - | - | 0.176 |
| 23RH | >20 | 4.2 | + | ++ | 0.453 |
| 23LH | 7.16 | 3.2 | +++ | + | 0.356 |
| 24LF | 0.38 | 1.6 | - | - | 0.298 |
| 25LF | 1.52 | 4 | + | - | 0.154 |
| 25RH | 0.58 | 1.6 | - | - | 0.12 |
| 25RF | 1.01 | 2.6 | + | - | 0.154 |
| 25LH | 1.37 | 1.8 | - | + | 0.179 |
| 26RH | 18.64 | 2.7 | +++ | + | 0.4 |
| 26LF | 18.39 | 2.3 | ++ | + | 0.41 |
| 27RF | 5.38 | 2.8 | +++ | ++ | 0.292 |
| 27LF | 5.56 | 2.5 | + | ++ | 0.3 |
| 27RH | 6.55 | 3.2 | + | ++ | 0.35 |
| 27LH | 0.52 | 3.1 | - | + | 0.13 |
| 28RH | 11.8 | 2.2 | + | ++ | 0.32 |
| 28LH | 2.02 | 2.5 | + | + | 0.156 |
| 28RF | 3.36 | 3.1 | + | ++ | 0.25 |
| 28LF | >20 | 2.4 | +++ | + | 0.45 |

SCC = somatic cell count; EC = electrical conductivity; CMT = California mastitis test; BTB = bromothymol blue; NAGase = *N*-acetyl-β-D-glucosaminidase
