# Supplementary figures and images for: Correction: Isolation, Biochemical and Molecular Identification, and In-Vitro Antimicrobial Resistance Patterns of Bacteria Isolated from Bubaline Subclinical Mastitis in South India
Source: PLoS One. 2015 Dec 23;10(12):e0145897. doi: 10.1371/journal.pone.0145897 (PMC4689394; doi:10.1371/journal.pone.0145897)

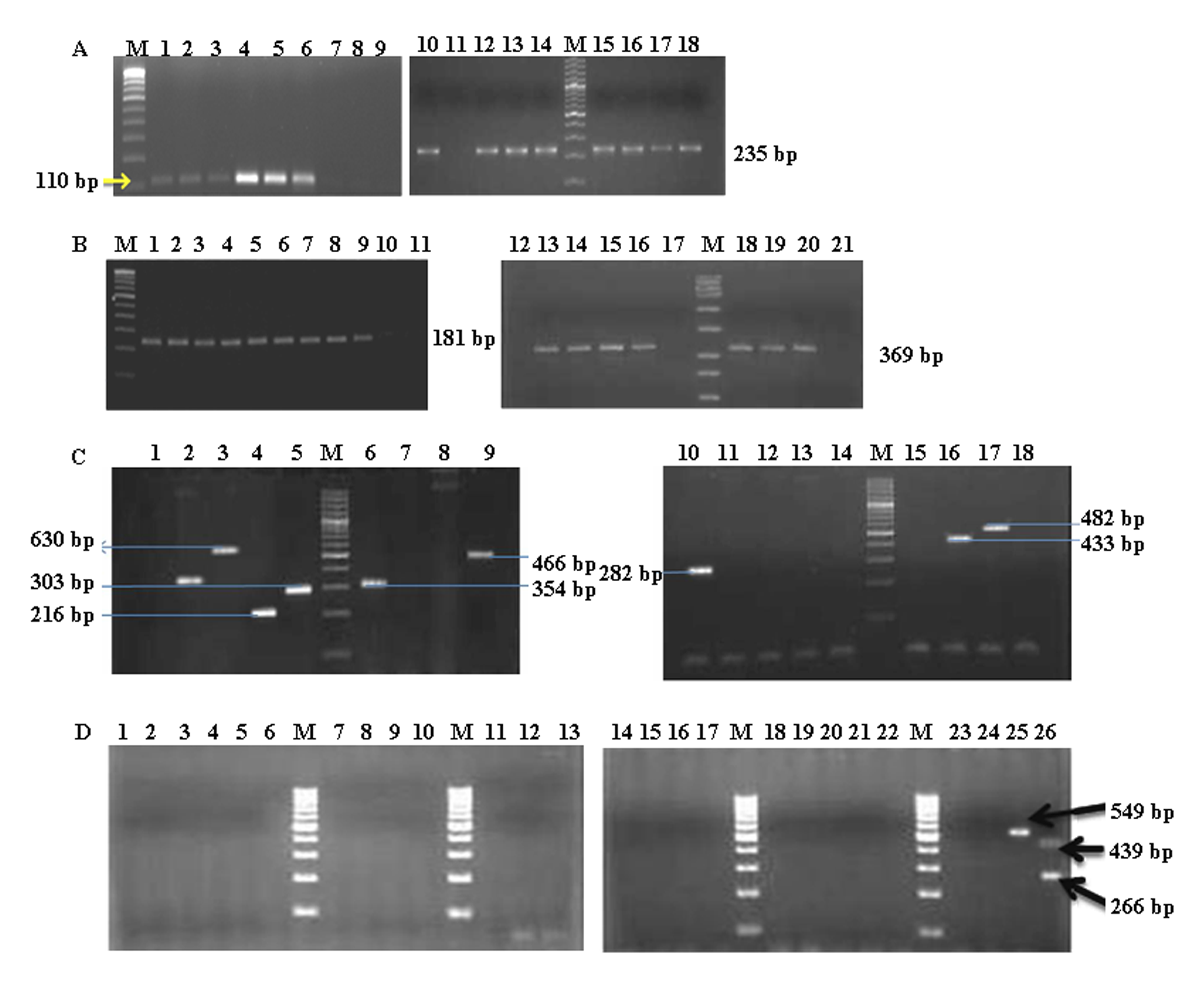

Supplement: S1 Fig — (TIF) [file pone.0145897.s001.tif]
